# Complete Mitochondrial Genome of *Piophila casei* (Diptera: Piophilidae): Genome Description and Phylogenetic Implications

**DOI:** 10.3390/genes14040883

**Published:** 2023-04-08

**Authors:** Shenghui Bi, Yanfei Song, Linggao Liu, Jing Wan, Ying Zhou, Qiujin Zhu, Jianfeng Liu

**Affiliations:** 1 School of Liquor and Food Engineering, Guizhou University, Guiyang 550025, Chinalgliu_0127@163.com (L.L.);; 2 Scientific Observing and Experimental Station of Crop Pest in Guiyang, Guizhou Provincial Key Laboratory for Agricultural Pest Management of the Mountainous Region, Institute of Entomology, Guizhou University, Ministry of Agriculture, Guiyang 550025, China; gs.songyf20@gzu.edu.cn

**Keywords:** Piophilidae, mitochondrial DNA, phylogenetic analysis, comparative analyses

## Abstract

*Piophila casei* is a flesh-feeding Diptera insect that adversely affects foodstuffs, such as dry-cured ham and cheese, and decaying human and animal carcasses. However, the unknown mitochondrial genome of *P. casei* can provide information on its genetic structure and phylogenetic position, which is of great significance to the research on its prevention and control. Therefore, we sequenced, annotated, and analyzed the previously unknown complete mitochondrial genome of *P. casei*. The complete mt genome of *P. casei* is a typical circular DNA, 15,785 bp in length, with a high A + T content of 76.6%. It contains 13 protein-coding genes (PCG), 2 ribosomal RNA (rRNA) genes, 22 transfer RNA (tRNA) genes, and 1 control region. Phylogenetic analysis of 25 Diptera species was conducted using Bayesian and maximum likelihood methods, and their divergence times were inferred. The comparison of the mt genomes from two morphologically similar insects *P. casei* and *Piophila megastigmata* indicates a divergence time of 7.28 MYA between these species. The study provides a reference for understanding the forensic medicine, taxonomy, and genetics of *P. casei*.

## 1. Introduction

*P. casei* (Linnaeus, 1758) (Diptera: Piophilidae) belongs to the holometabolous insects, and its growth cycle includes four stages: egg, larva, pupa, and adult. The life cycle of *P. casei* lasts 12–30 days, and it can usually breed 7–9 generations per year [1]. *P. casei* exhibits a highly developed olfactory system, displaying a specific attraction to fishy and putrid odors [2,3], and has been identified as the most harmful pest to dry-cured ham. Its voracious appetite enables it to quickly consume ham, leading to the formation of cavities, darkening of the flesh, and the production of a putrid smell, ultimately resulting in significant damage to ham quality and considerable economic losses for ham manufacturers [4]. Similarly, it has been found in cheese and fish, as well as in human corpses in advanced stages of decomposition. It is widely distributed in the world and has been documented not only in Guizhou, Yunnan, and Zhejiang provinces in China but also in many parts of Europe and North America [5]. In Italy, *P. casei* is used to make cheese products with a unique flavor [6]. However, ingestion of the larvae by humans can lead to intestinal myiasis, so safety remains a concern [7,8].

*P. casei* is generally not favored in the food industry, but it is highly valued in the fields of forensic medicine and forensic entomology. In forensic medicine, the Piophilidae family is extremely important, especially in the advanced stages of decomposition, as it can provide valuable information when determining the minimum post-mortem interval [5,9].

Piophilidae is a small family of flies that comprises several species, including *P. casei* and *Piophila megastigmata* (McAlpine 1978) [10,11]. In the early years, the high similarity in appearance between *P. casei* and *P. megastigmata*, coupled with the lack of research on *P. megastigmata*, made it challenging to differentiate between the two species, with *P. megastigmata* often being misidentified as *P. casei*. Until recent times, *P. megastigmata* was found to be more prevalent on cadavers than *P. casei* [12]. However, the genetic information available on *P. casei* is still limited, and forensic entomologists rely mostly on morphological observations rather than genetic comparisons to distinguish and compare the appearance of *P. casei* and *P. megastigmata* [13,14]. 

Mitochondria (mt) genes are powerful molecular markers in phylogenetic and population genetics studies. The complete mt genomes that contain more variants provide more information than partial mt gene sequences to help scientists resolve phylogenetic relationships and infer species evolution [15,16,17]. The mt genome of insects and mites generally contains 13 protein genes, 22 tRNAs, 2 rRNAs, and a control region [18,19,20]. In this study, the complete mt genome was assembled, and the basic characteristics of *P. casei* mt genes were studied, focusing on the comparison of the PCGs between *P. casei* and *P. megastigmata*. The known mt genome data of 25 Diptera insects from GenBank was selected for the construction of phylogenetic relations and evolution time, laying the foundation for a more in-depth study at the molecular level and providing theoretical bases and ideas for the accurate identification of forensic insects *P. casei* and *P. megastigmata*.

## 2. Materials and Methods

### 2.1. Sample Collection

Fifty adult specimens of *P. casei* were collected on 2 November 2021 from the dry-cured hams in Panxian (25°75.87′ N; 104°52.57′ E), Guizhou Province, China. The specimens were preserved in 100% alcohol for species identification and DNA extraction in the laboratory. Illustrations of *P. casei* were obtained from samples of eggs, larvae, pupae, and adults that were collected. Color images were captured using the Keyence VHX-6000 model (Keyence, Osaka, Japan) color-image analysis instrument.

### 2.2. DNA Extraction, Library Construction, and Sequencing

Genomic DNA was extracted from *P. casei* male adults using Qiagen DNeasy Blood and Tissue Kit (Qiagen, Hilden, Germany) according to the manufacturer’s instructions. DNA degradation and contamination were monitored on 1% agarose gels. DNA purity was measured using a NanoDrop 2000 spectrophotometer (Thermo Fisher Scientific, Waltham, MA, USA), and DNA was quantified using Qubit DNA Assay Kit Fluorometer (Life Technologies, Carlsbad, CA, USA).

The Truseq Nano DNA HT Sample Preparation Kit (Illumina, San Diego, CA, USA) was used to generate sequencing libraries following the manufacturer’s instructions, and index codes were added to assign the sequences to each sample. The DNA sample (1.5 µg DNA per sample) was fragmented by sonication to a size of 350 b. Then the DNA fragments were end-polished, A-tailed, and ligated to the full-length adapter from the sequencing kit with further PCR amplification. Last, the PCR products were purified (AMPure XP system) and analyzed for size distribution by an Agilent2100 Bioanalyzer. These constructed libraries were sequenced with the Illumina NovaSeq 6000 platform (BIOZERON, Shanghai, China), and 150 bp paired-end reads were generated with an insert size of around 350 bp [21].

### 2.3. Genome Assembly and Gene Annotation

In this study, a script implemented in MitoZ was utilized to filter the extracted mitochondrial gene sequences, which effectively removed reads containing numerous ‘N’s, low-quality reads, or PCR duplicates (i.e., pairs of identical reads) [22]. Then the mt genome was assembled in MitoZ using De novo assembly. De novo assembly is an algorithm well suited to mt mitochondrial genome assembly, which, with the assistance of other algorithms and evaluation metrics in MitoZ, enables better assembly of genomic sequences [23]. The principle is that the average sequencing depth of mitochondrial genome reads is much higher than that of the nuclear genome, and different Kmer parameters are set to achieve the best possible assembly. The Kmer parameter was finally adjusted to 33 to achieve the best assembly results. Protein-coding genes were annotated using scripts in MitoZ. tBLAST in BLAST v2.2.19 [24] was used to find candidate PCG sequences by matching sequences to the protein database sequences (MitoZ has a built-in database of insect PCG annotations); Genewise v2.2.0 [25], which produces faster and more accurate results, was used to identify each PCG for annotation. tRNAs were annotated using MiTFi [26] of the covariance model (CM) for annotation, and rRNAs were annotated using infernal-1.1.1 [27] and rRNA CMs based on an extensive manually curated alignment [28]. The complete mitogenome sequence of *P. casei* has been submitted to GenBank (ON204020).

### 2.4. Genomic and Phylogenetic Analysis

The circular map of the *P. casei* mt genome was painted using proksee (https://proksee.ca/ (accessed on 10 September 2022)). The basic information about the genome was calculated using CodonW v1.4.2. The relative synonymous codon usage (RSCU) values were analyzed using ggplot2 v3.3.6 and plotted using aplot v0.1.6 packages in the R v4.0.4 [29].

To understand the taxonomic position of *P. casei* in Diptera, the mt genomes of 25 insects (including 2 outgroup mt genomes) were downloaded from GenBank and used to construct phylogenetic trees using both the maximum likelihood (ML) and Bayesian inference (BI) methods. The nucleotide diversity (Pi), the nonsynonymous substitution rate (Ka), and the synonymous substitution rate (Ks) were calculated using Launch DnaSP6 v6.12.3 [30], and the results were plotted using GraphPad Prism v8.0.2. Base sequence substitution saturation was analyzed and plotted using DAMBE v7.3.11 [31].

The 22 tRNA genes, 13 PCGs, and 2 rRNA genes of each of the 25 insects were extracted from NCBI and imported into PhyloSuite v1.2.2 [32]. The sequences were trimmed using Gblocks v0.91 b [33] to remove redundant codons and then concatenated using concatenate sequence to form a single concatenated sequence. All sequences were then aligned using MAFFT v7.313 and optimized using MACSE v2.0.1 [34]. The secondary structure of 22 tRNAs was predicted with ARWEN and compared manually. The MFE (minimum free energy) structures of two ribosomal RNA genes (rrnL and rrnS) were obtained by RNAfold [35] prediction. For the tandem sequences, the optimal GTR + F + I + G4 and GTR + F + G4 molecular phylogenetic model was found using ModelFinder [36] and imported into MrBayes v3.2.6 [37] and IQ-TREE v1.6.8 [38] for BI and ML phylogenetic tree construction with 104 and 2 × 106 bootstraps, respectively. iTol (https://itol.embl.de/itol.cgi (accessed on 10 September 2022)) was used to visualize the phylogenetic trees, and then the developmental trees constructed by the two methods were compared.

### 2.5. Divergence Time Estimate

The divergence time was estimated from the BI phylogenetic trees with timetree5 (http://www.timetree.org/ (accessed on 10 September 2022)) and BEAST v1.10.4 [39] according to the related literature and using age 70 MYA as the divergence time calibration [40]. The estimation of divergence models for subfamilies was based on complete mt genomes according to the strict clock log-normal model in BEAST. A calibrated Yule model was used and spliced using the GTR + F + I + G4 and GTR + F + G4 models. After confirming the convergence of the chains with Trancer v1.7.2 [41], the first 10% of generations were burn-in as ageing every 1000 generations sampled at 10^7^ conditions. We summarized the subsample trees in a maximum clade credibility tree with mean heights using Tree Annotator v1.10.4. The mean heights and 95% highest probability density (95% HPD) were displayed in Figtree v1.4.3. The labeling and photo-compositing of the images in this article were made using Adobe Photoshop 2022 and Adobe Illustrator 2022.

## 3. Results and Discussion

### 3.1. General Features of P. casei mt Genome

**Figure 1:**
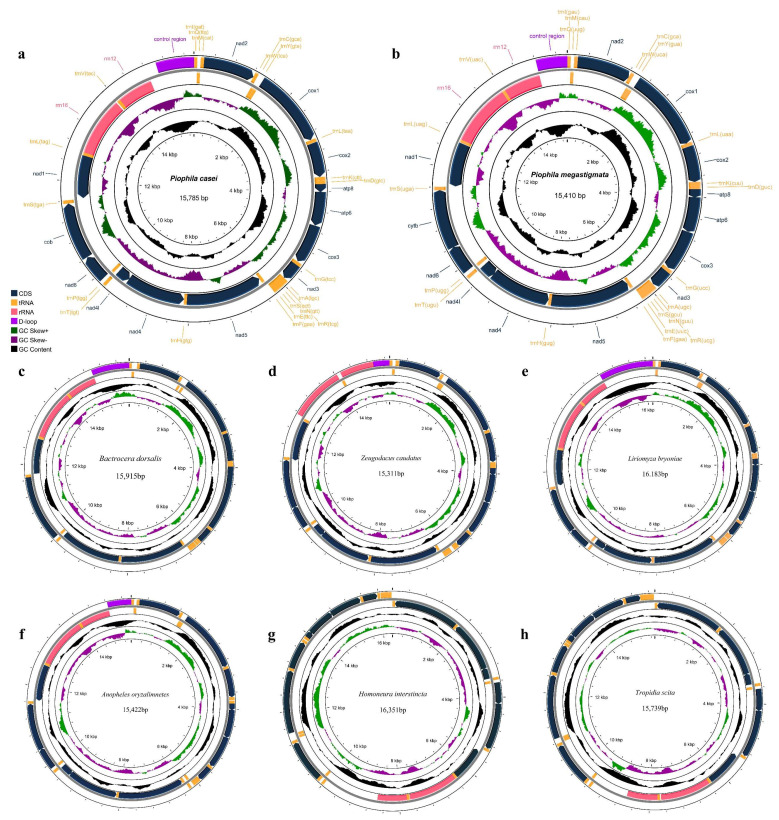
The mt genome of eight species. (**a**) *P. casei*, (**b**) *P. megastigmata*, (**c**) *B. dorsalis*, (**d**) *Z. caudatus*, (**e**) *L. bryoniae*, (**f**) *A. oryzalimnetes*, (**g**) *H. interstincta*, and (**h**) *T. scita*. The chain is marked with an arrow indicating the direction of gene transcription. Gene lengths correspond to nucleotide lengths in the diagram. The outermost layer is the J chain, and the second layer is the N chain. The third black peak indicates GC content, and the purple and green peaks indicate the ±skew of GC.

The complete mitochondrial genome of *P. casei* is 15,785 bp long (GenBank No. ON204020) and contains 13 PCGs (Protein-coding genes) (11,206 bp), 22 tRNA genes (1467 bp), 2 rRNA genes (2107 bp), and 1 control region (744 bp). The circular map of the *P. casei* mt genome is shown in Figure 1a and is generally consistent with the mt genomes of eight other Diptera, such as *P. megastigmata* (15,410 bp) (Figure 1b), *Bactrocera dorsalis* (15,915 bp) (Figure 1c), *Liriomyza bryoniae* (16,183 bp) (Figure 1e), and *Anopheles oryzalimnetes* (15,422bp) (Figure 1f). The difference in mt genome length between these five species is mainly due to the differences in the size of the control region (from a minimum of 388bp to a maximum of 1354 bp), as previously observed between Diptera species [42]. In Diptera, the mt genomes of some species, such as *Zeugodacus caudatus* (15,311 bp) (Figure 1d), *Homoneura interstincta* (16,351 bp) (Figure 1g), and *Tropidia scita* (15,739 bp) (Figure 1f) are arranged differently than most species.

The mitochondrial genomes of *P. casei* and *P. megastigmata* exhibit a high degree of conservation, as evidenced by the presence of 37 genes in each genome, as well as the absence of any inversion events or swapping of gene coding directions.

In terms of ATCG content and AT-skew, CG-skew, the mt genome of *P. megastigmata* is only slightly different than that of *P. casei* (Table 1). The difference between the two is more pronounced in the AT% and AT-skew of the tRNA gene, indicating differences in amino acid content between the two species.

The complete *P. casei* mt genome consisted of 39.5% A, 37.1% T, 13.8% C, and 9.6% G. The total A + T content was 76.6%, which is relatively high among Tephritidae species. The mt genome had a positive AT skew (0.03) and a negative CG skew (−0.18); according to the previous reports, a bias against the use of Gs in all strands is characteristic of the metazoan mitochondrial genome [43]. Four PCGs (*nad5*, *nad4*, *nad4l*, *nad1*), eight tRNA genes (*trnQ*, *trnC*, *trnY*, *trnF*, *trnH*, *trnP*, *trnL*, *trnV*), and two rRNA genes (*rrn12*, *rrn16*) were found on the minority strand (N strand), while the remaining genes were on the majority strand (J strand) (Table 2). Ten genes overlap on the mt genome of *P. casei*, with a total of thirty-four overlapping bases. There are 14 gene spacers, with the largest spacer (16 bp) occurring between rrn12 and the control region.

**Figure 2:**
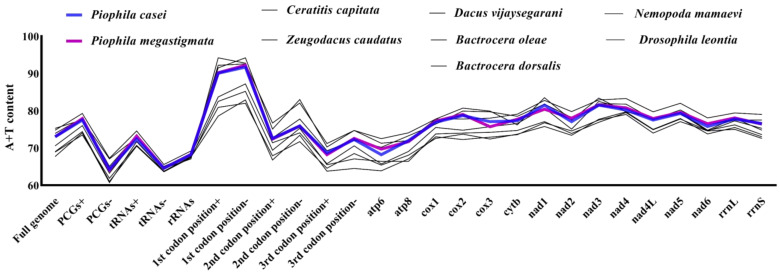
AT content of genes in various parts of the mt genome among the nine species in Tephritoidea.

In order to compare the AT% variations among different species within the same family, nine species in Tephritoidea were selected [41]. Overall, the AT content of each of their genes was found to be similar, with significant differences only in the proportion of codons used in the first, second, or third codons. The first codon position in both *P. casei* and *P. megastigmata* was found to have higher AT% compared with several other species in the Tephritoidea(Figure 2).

### 3.2. Protein-CODING Genes and Codons

The PCGs accounted for 71% (11,206 bp) of *P. casei* mt genes. The start codon ATN was found in 11 of the 13 PCGs genes, including ATG in *cox2*, *atp6*, *cox3*, *nad4*, *nad4l*, and *cytb*; ATT in *nad2*, *atp8*, *nad3*, and *nad6*; and ATA in *nad1*. However, *cox1* uses CGA and *nad5* uses GTG as their start codon. The stop codons of 13 PCGs are mostly TAA (*nad2*, *cox1*, *atp8*, *atp6*, *cox3*, *nad3*, *nad4l*, *nad6*) [44] and include TAG (*cytb*) and T (*cox2*, *nad5*, *nad4*, *nad1*). The PCGs of the two species were measured to have only 863 different loci.

**Figure 3:**
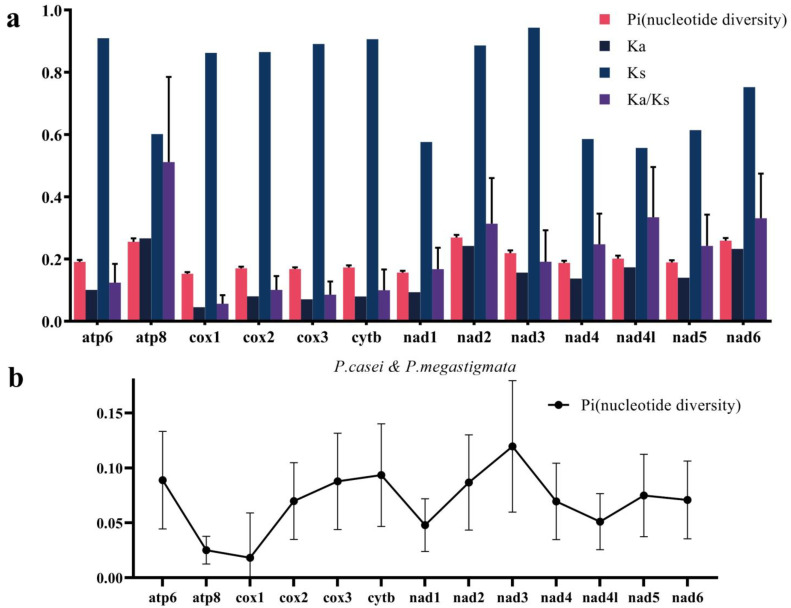
(**a**) Nucleotide diversity (Pi) values of *P. casei* and *P. megastigmata* PCGs; (**b**) Pi values and Ka/Ks values of PCGs in the mt genomes of 26 species.

The nucleotide diversity (Pi) was compared for the PCGs of 26 species (Figure 3a), and the strength of polymorphisms was used to characterize the PCGs of 26 species. Overall, the Pi values ranged from 0.153 to 0.269. The *nad2* (Pi = 0.269) gene had the greatest variability among these genes, followed by *nad6* (Pi = 0.259), *atp8* (Pi = 0.255), *nad4l* (Pi = 0.202), and, finally, *cox1* (Pi = 0.153) with the least variability. It shows that *cox1* has high genetic stability among dipterans, while *nad2* has weak stability and is suitable for the study of the evolution of variation [45]. The Pi values of *P. casei* and *P. megastigmata* were also compared individually in each species (Figure 3b): *cox1* (Pi = 0.018) demonstrated the least variability, *nad3* (Pi = 0.120) showed the greatest variability, and *atp8* (Pi = 0.025) had a low degree of variability, unlike the results of the multispecies comparison.

The frequency of non-synonymous mutations (Ka), the frequency of synonymous mutations (Ks), and the ratio of the two (Ka/Ks) were calculated for 13 PCGs of 26 species and used to understand the rate at which synonymous and non-synonymous mutations occur (Figure 3d). The Ka/Ks values determine whether there is selection pressure on the PCGs and also the degree of conservation of the coding gene. The closer the Ka/Ks is to 1, the lower the selection pressure is on that gene. The Ka/Ks values range from 0.0561 to 0.5119, and the order of Ka/Ks values for PCGs from the smallest to the largest is as follows: *cox1*, *cox3*, *cytb*, *cox2*, *atp6*, *nad1*, *nad3*, *nad5*, *nad4*, *nad2*, *nad6*, *nad4l*, *atp8*. Among them, the genes of the cytochrome oxidase (cox) family *cox1*, *cox2*, *cox3*, and *cytb* have smaller Ka/Ks values, indicating that they face a stronger purifying selection, a slower evolutionary rate, especially *cox1*, which, because of the lowest Ka/Ks value, has been subjected to the highest purity selection. This may be why it has been used for the study of evolutionary biology as the molecular marker. Combined with Pi and Ka/Ks values, the evolutionary rate and variability of *cox1* are very low. Based on the computational information, the molecular weight (Da), isoelectric points, instability index, aliphatic index, GRAVY, and other indices of the proteins were predicted (Table 2).

The Pi and Ka/Ks values of *atp8* are high compared with other genes. In contrast, another atp synthase *atp6* shows a much slower evolutionary rate. The genes *atp6* and *atp8* are arranged in close proximity and overlap by 7 bp, indicating that *ap6* and *atp8* did not co-evolve as a gene cluster during the evolutionary process. The Pi of *atp8* within the dipteran range showed a large difference in Pi values between the two species, and the difference between the two results is a better indication that the two species share similarities in an evolutionary direction.

**Figure 4:**
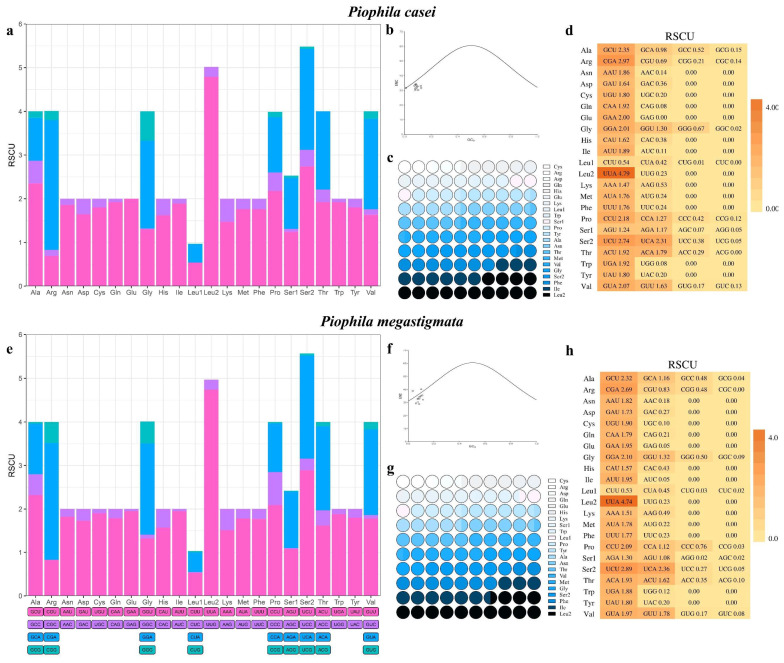
RSCU information of PCGs (**a**) Relative synonymous codon usage (RSCU) in the mtgenomes of *P. casei*; (**b**) ENc-plot of *P. casei*; (**c**) Amino acid usage graph for *P. casei*; (**d**) Heat map of RSCU results for *P. casei* calculated by CodonW software; (**e**) Relative synonymous codon usage (RSCU) in the mtgenomes of *P. megastigmata*; (**f**) ENc-plot of *P. megastigmata*; (**g**) Amino acid usage graph for *P. megastigmata*; (**h**) Heat map of RSCU results for *P. megastigmata* calculated by CodonW software.

The 13 PCGs were further analyzed by calculating the effective codon number (ENc) (Figure 4b), with values closer to 20 indicating a higher expression and a greater codon preference. The GC of the silent third codon posit (GC3s) value was calculated to reflect the probability of synonymous codon use. Plotting the ENc-plot according to the formula Enc = 2 + GC3 + 29/(GC32 + (1 − GC3)2) with the ENc value as the vertical axis and GC3 as the horizontal axis revealed that most of the codons were distributed around the standard curve (Figure 4b). The results suggest that the formation of codon bias in the *P. casei* mt genome may be related to mutations alone and is less affected by other factors (e.g., natural selection) [46]. In comparison, *P. megastigmata* is more subject to external factors that produce codon bias (Figure 4f).

The number of synonymous codons (L_sym) and the total number of amino acids (L_aa) were analyzed [47]. Based on the computational information, the molecular weight (Da), isoelectric points, instability index, aliphatic index, GRAVY, and other indices of the proteins were predicted. The results show that *cox1*, *cox2*, *cox3*, and *cytb* are structurally stable and can be used for species classification and mutation-related studies (Table 3), which is consistent with the Pi values as well as with the Ka/Ks values analysis [48].

In *P. casei*, the five codons with the highest relative synonymous codon usage rate (RSCU) [49] are UUA (4.79), CGA (2.97), UCU (2.74), GCU (2.35), and UCA (2.31) (Figure 4a,d). The RSCU values of these codons are all greater than one, indicating that they are more frequently used in encoding amino acids. The RSCU of *P. casei* and *P. megastigmata* show a similar bias, with only minor differences in the use of a few codons, such as CGC, CUC, and ACG. As with other Diptera, the mt genome of Piophilidae is more biased toward the use of amino acids encoded by codons with A or T in the third position [42]. The amino acid use frequencies of PCGs in the mt genomes of *P. casei* and *P. megastigmata* were counted and showed that the number of 20 amino acids varied, but both had the highest frequency of leucine (Leu) usage, followed by isoleucine (Ile), serine (Ser), and glycine (Gly) (Figure 4c,g). Overall, there is still a predominance of nonpolar amino acids (hydrophobic) and very little amount of basic amino acids (Argue, Lysol, His), with no bias toward acidic (Asp, Glu). By comparing the amino acid content with the codon, it was found that the codon content with the third codon being A or G had an effect on the amino acid content. The small difference in the frequency of amino acid use between the two species suggests that there is also a bias toward the use of amino acids.

### 3.3. Transfer and Ribosomal RNA Genes

**Figure 5:**
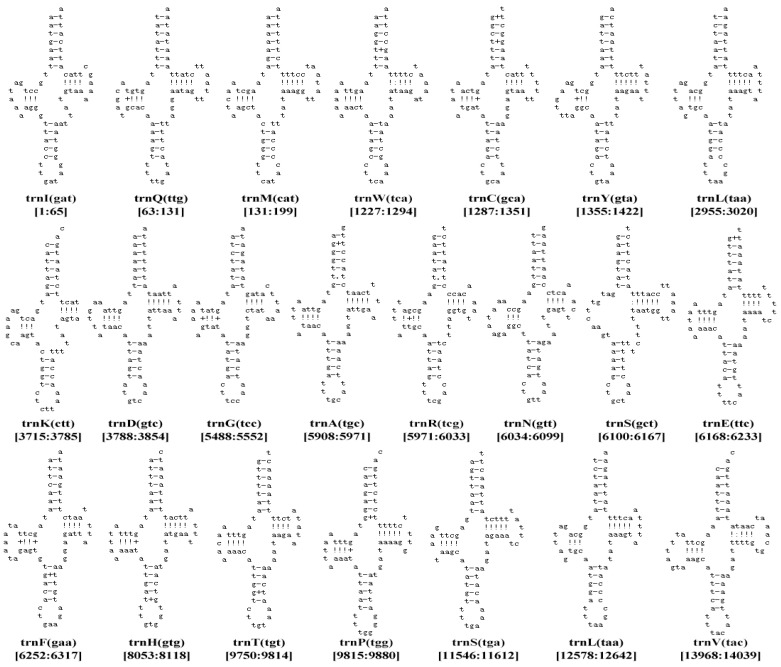
The 22 tRNA secondary structures of *P. casei*. The numbers below indicate their sequence position in the mt genome.

**Figure 6:**
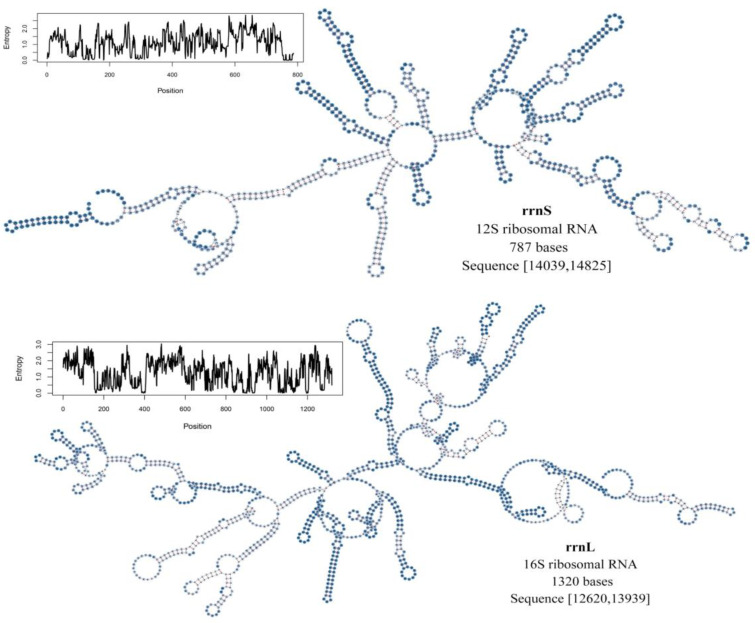
Secondary structure of rrnS and rrnL mt genome of *P. casei*.

The complete mt genome of *P. casei* included 22 tRNA genes and 2 rRNA genes. The 22 tRNAs had a length between 65 bp and 72 bp. A total of 21 typical trilobal secondary structures were predicted. The tRNA gene trnS was the only one missing the D-arm (Figure 5). It is very common in the fly family, and the absence of this arm does not affect the function of the tRNA genes such as *Chrysomya chloropyga* [43]. The predicted secondary structure maps of rrnL and rrnS by energy-minimization (MFE) are in general agreement with the secondary structures proposed for other insects and provide a reference for subsequent studies (Figure 6) [19,50,51]. The features of tRNAs were conserved between the *P. casei* and *P. megastigmata* genomes. The 16s-rRNA gene is 1320 bp and is located between trnL and trnV. The 12s-rRNA gene is 787 bp and is located between trnV and the control region. The complete mt genome of *P. casei* has a 744 bp control region, which is responsible for regulating DNA replication and transcription. The A + T content of this region is 92.61% between rrnS and trnL.

### 3.4. Phylogenetic Analysis

**Figure 7:**
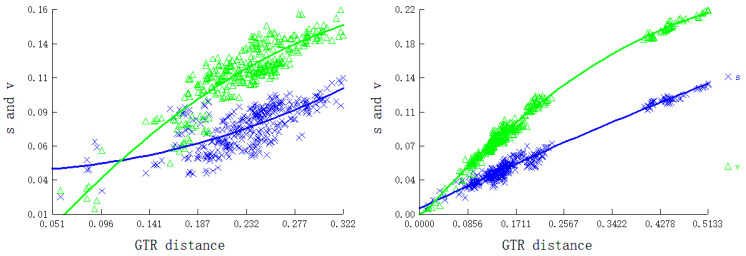
Substitution saturation plots of mt genomic PCGs and RNAs for 26 species. Plots in blue and green indicate transition and transversion, respectively.

We used the complete mt genome sequence of *P. casei* for phylogenetic analysis, starting with a tree-building assessment of all species’ gene sequences (PCGs and RNAs). The saturation plots in Figure 7 show the relationships between general time reversible (GTR) distance and transition/transversion rates. No significant substitution saturation was detected for any codon positions of the 13 PCGs and RNAs in either the symmetrical or asymmetrical topology tests (Iss (0.2701, 0.3261) < Iss.c (0.8452, 0.8046), *p* < 0.001) [52]. The Iss values were all lower than the Iss.c values, indicating that the results of the tree construction using the complete mt genome of 26 species were meaningful for phylogenetic tree construction. Therefore, we subsequently used the complete mt genomes of 26 species for phylogenetic tree construction and analysis.

**Figure 8:**
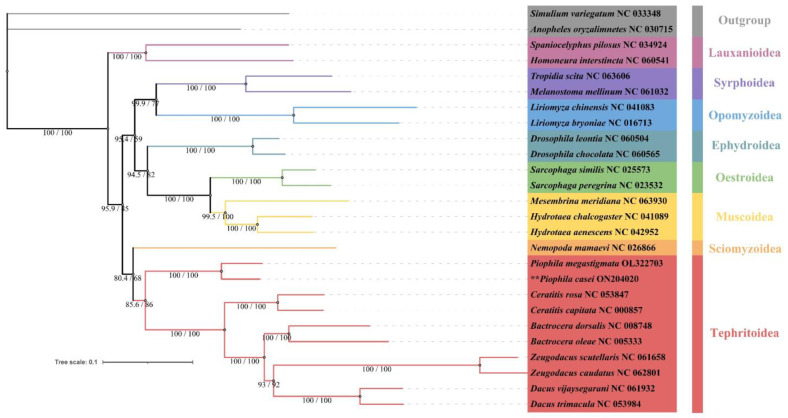
A genome-wide phylogenetic tree of the mitochondria of 26 dipteran insect species constructed based on ML analysis. Two values of developmental dendrites (SH-aLRT support/bootstrap support). (The specie marked with ** is the main subjects of study in this research).

**Figure 9:**
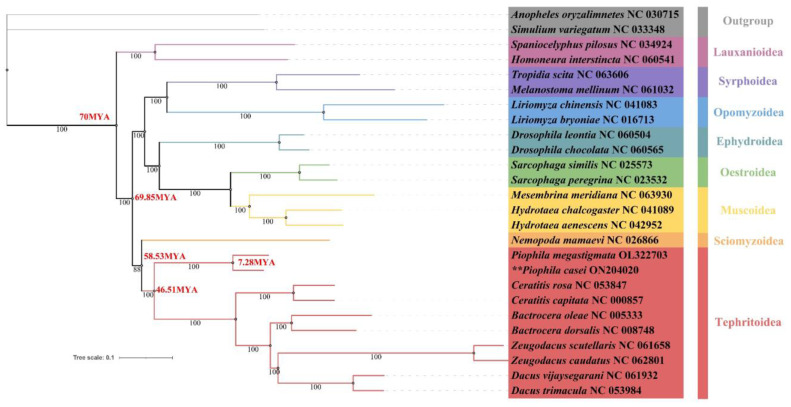
A genome-wide phylogenetic tree of the mitochondria of 26 dipteran insect species constructed based on BI analysis. The red values in the developmental tree indicate the time of divergence between species MYA (million years ago). (The specie marked with ** is the main subjects of study in this research).

In this study, phylogenetic trees were constructed using 12 PCG genes, 2 rRNA genes, and 22 tRNA genes from 26 species using different best-fit substitution models, the ML algorithm (Figure 8), and the BI algorithm (Figure 9). Two common outgroups (Culicoidea, *A. oryzalimnetes*, NC_030715, and Chironomoidea, *Simulium variegatum*, NC_033348) were used. Both ML and BI phylogenetic analyses produced a similar topology with slightly different node support values. The node support was generally higher for the BI tree than for the ML tree, which is common in studies of taxa [53,54,55]. High support was obtained throughout the phylogenetic tree, with Tephritoidea and Sciomyzoidea appearing distinctly separated from other families, and the abdomen of species within Tephritoidea is consistent with Hoi-Sen’s study [56].

We did not use the older calibration points (238.5–295.4 MYA) and instead obtained divergence time data that is more realistic [40]. Combined with the timing of divergence, the evolutionary history of Oestroidea species and Piophilidae species differ significantly, although they share similar feeding habits, with their ancestors diverging as early as 69.85 MYA (95% HPD: 68.69–70.89 MYA) around the late Cretaceous periods. The Piophilidae of the Tephritoidea diverged from other plant-feeding species at 46.51 MYA (95% HPD: 37.16–55.81 MYA) to produce two different diets, despite sharing similar morphological characters.

The results of the phylogenetic tree show that *P. casei* is very closely related to *P. megastigmata*; the mt genetic distance within the same family of 26 Piophilidae species is 0.062. Judging by the evolutionary branch lengths, the *P. megastigmata* has longer branch lengths, reflecting its greater degree of genetic variation and a higher degree of evolution. The divergence between *P. casei* and *P. megastigmata* was inferred at 7.28 MYA (95% HPD: 3.64–18.20 MYA) around the late Cenozoic Tertiary during the Miocene [42]. These results may provide a valuable basis for the study of the evolution of the *P. casei* gene family, population transmission, and biodefense potential [57,58,59].

## 4. Conclusions

Previous studies have attempted to describe the phylogenetic relationships of *P. casei* and to identify it taxonomically based on morphological characters or nucleotide fragments. In this study, we report the first complete mt genome of *P. casei* and document the phylogenetic relationships of *P. casei* with the mt genomes of other 26 fly species. This research provides the evidence for the accurate identification of two morphologically closely related forensic insects *P. casei* and *P. megastigmata*, and a theoretical basis for a more in-depth study of their genes. Unfortunately, the sample size of the species used in the article is limited, and further investigations are needed to compare the mitochondrial genome among families of Diptera as a whole, as well as to explore the species divergence time. A deeper exploration of the *P. casei* genome also merits further investigation.

## Figures and Tables

**Table 1:** The base composition of the complete mt genome of *P. casei* and *P. megastigmata*.

**(*P. casei*)** **Region**	**Size (bp)**	**A%**	**T%**	**AT-skew**	**C%**	**G%**	**CG-skew**
Mitogenome	15,785	39.5	37.1	0.03	13.8	9.6	−0.18
Protein-coding genes	11,206	31.2	43.6	−0.17	12.3	12.9	0.02
tRNA	1467	37.7	38.9	−0.02	10.2	13.2	0.13
rRNA	2107	38.2	41.0	−0.04	7.4	13.4	0.29
**(*P. megastigmata*)** **Region**	**Size (bp)**	**A%**	**T%**	**AT-skew**	**C%**	**G%**	**CG-skew**
Mitogenome	15,410	39.2	37.2	0.03	14.1	9.5	−0.2
Protein-coding genes	11,207	31.3	43.6	−0.16	12.4	12.7	0.01
tRNA	1463	38.3	38.8	−0.01	10.0	12.9	0.13
rRNA	2111	37.9	41.8	−0.05	7.1	13.2	0.3

A%, T%, C%, and G% represent the proportion of the respective nucleotides in the complete mitochondrial genome.

**Table 2:** Gene order and basic characteristics of the *P. casei* mt genome.

Gene	Type	Strand	Position (Start–End)	Length (bp)	Intergenic Spacer
trnl(gat)	tRNA	J	1–65	65	−
trnQ(ttq)	tRNA	N	63–131	69	−3
trnM(cat)	tRNA	J	131–199	69	−1
nad2	CDS	J	200–1228	1029	0
trnW(tca)	tRNA	J	1227–1294	68	−2
trnC(gca)	tRNA	N	1287–1351	65	−8
trnY(gta)	tRNA	N	1355–1422	68	3
cox1	CDS	J	1424–2954	1531	1
trnL(taa)	tRNA	J	2955–3020	66	0
cix2	CDS	J	3027–3714	688	6
trnK(ctt)	tRNA	J	3715–3785	71	0
trnD(gtc)	tRNA	J	3788–3854	67	2
atp8	CDS	J	3855–4016	162	0
atp6	CDS	J	4010–4687	678	−7
cox3	CDS	J	4691–5479	789	3
trnG(tcc)	tRNA	J	5488–5552	65	8
nad3	CDS	J	5553–5906	63	0
trnA(tgc)	tRNA	J	5908–5971	66	1
trnR(tcg)	tRNA	J	5971–6033	68	−1
trnN(gtt)	tRNA	J	6034–6099	66	0
trnS(qct)	tRNA	J	6100–6167	68	0
trnE(ttc)	tRNA	J	6168–6233	66	0
trnF(gaa)	tRNA	N	6252–6317	66	18
nad5	CDS	N	6318–8052	1753	0
trnH(gtg)	tRNA	N	8053–8118	66	0
nad4	CDS	N	8119–9457	1339	0
nad4l	CDS	N	9451–9747	297	−7
trnT(tgt)	tRNA	J	9750–9814	65	2
trnP(tgg)	tRNA	N	9815–9880	66	0
nad6	CDS	J	9883–10,407	525	2
cytb	CDS	J	10,411–11,547	1137	3
trnS(tga)	tRNA	J	11,546–11,612	67	−2
nad1	CDS	N	11,628–12,576	949	5
trnL(tag)	tRNA	N	12,578–12,642	65	1
rrn16	rRNA	N	12,643–13,939	1297	0
trnV(tac)	tRNA	N	13,968–14,039	72	−2
rrn12	rRNA	N	14,039–14,825	787	−1
control region	D-loop	J	15,042–15,785	744	16

**Table 3:** Basic information on the protein molecules of the PCGs gene in the complete mt genome of *P. casei*.

Gene	ENc	GC3s	L_sym	L_aa	Molecular Weight (Da)	Isoelectric Points	Instability Index	Aliphatic Index	GRAVY
atp6	33.95	0.083	218	220	25,145.94	7.09	41.84	123.91	0.884
atp8	32.74	0.08	50	50	6118.28	9.4	44.61	93.77	0.477
cytb	32.52	0.083	363	365	43,057.3	8.33	29	126.64	0.781
cox1	33.09	0.101	496	496	56,373.37	5.97	26.74	110.69	0.732
cox2	34.23	0.077	221	222	26,113.28	4.92	31.38	113.64	0.346
cox3	33.01	0.114	246	250	30,025.75	6.2	32.14	98.21	0.516
nad1	31.27	0.082	305	308	36,067.56	8.45	32.83	127.06	1.085
nad2	33.72	0.127	332	333	39,520.5	9.1	39.9	121.78	0.924
nad3	31.66	0.105	114	114	13,510.31	5.66	45.26	134.19	0.998
nad4	30.26	0.061	426	436	51,075.7	8.3	38.68	127.65	1.068
nad4l	30.22	0.000	96	97	11,502.86	5.99	24.97	121.22	1.093
nad5	31.64	0.089	560	564	65,550.86	6.5	33.48	122.94	0.972
nad6	31.64	0.076	172	173	20,277.72	8.64	26.59	132.82	1.032

## Data Availability

The data presented in this study are available in the article and in the Supplementary Materials.

## Supplementary Materials

The following supporting information can be downloaded at: https://www.mdpi.com/article/10.3390/genes14040883/s1, Table S1: The optimal partition schemes and the best-fit replacement models for the Bayesian Inference (BI) and the maximum likelihood (ML); Figure S1: Genetic distances of PCGs between 26 Diptera species; Figure S2: Genetic distances of mt genomes between 26 Diptera species; Figure S3: AT-Skew and CG-Skew values for the mt genomes of 26 Diptera species; Figure S4: Box-and-whisker plots for nucleotide composition of each gene. (a) GC-skew; (b) AT-skew; Table S2. Divergence time estimates calculate information such as the ESS of the resulting value; Table S3. Summary of the representative species and their mitogenome information in this study. Figure S5. The life cycle states of *P. casei*.
