# Supplementary material for: Complete Mitochondrial Genome of Piophila casei (Diptera: Piophilidae): Genome Description and Phylogenetic Implications
Source: Genes (Basel). 2023 Apr 8;14(4):883. doi: 10.3390/genes14040883 (PMC10137744; doi:10.3390/genes14040883)
Supplement: Supplementary file 1 [file genes-14-00883-s001.zip › genes-2308108-supplementary.pdf]

Table S1. The optimal partition schemes and the best-fit replacement models for the Bayesian Inference (BI) and the Maximum Likelihood (ML)

Partitions	Models	Gene
P1	GTR+F+I+G4	trnA, trnC, trnF, trnG, trnH, trnI, trnK, trnL1, trnL2, trnM, trnN, trnP, trnQ, trnR, trnS1, trnS2, trnV, trnW, trnY
P2	GTR+F+G4	rrnL, rrnS
P3	GTR+F+I+G4	trnD, trnE, trnT, atp6, nad3
P4	GTR+F+I+G4	atp8, nad2, nad6
P5	GTR+F+I+G4	cox1, cox2, cox3, cytb
P6	GTR+F+I+G4	nad1, nad4L, nad4, nad5

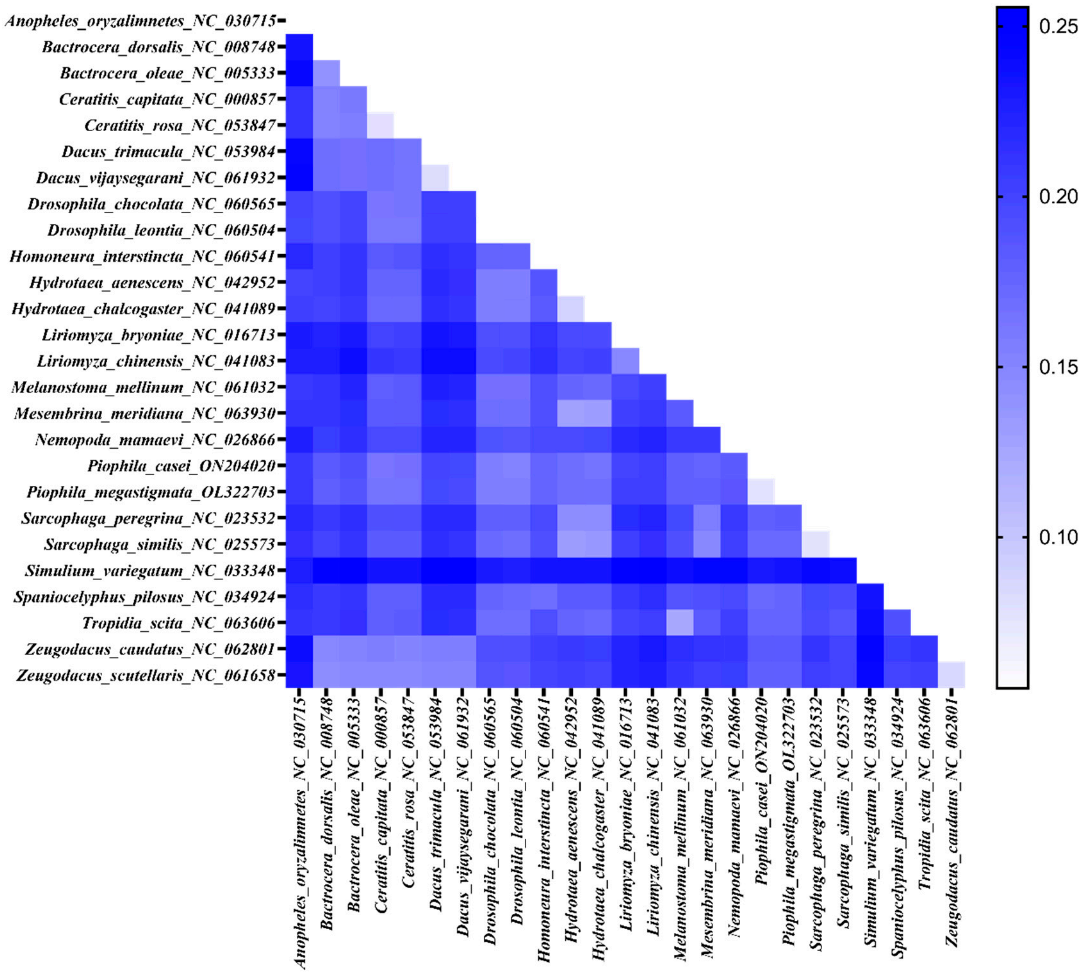

Figure S1. Genetic distances of PCGs between 26 Diptera species.

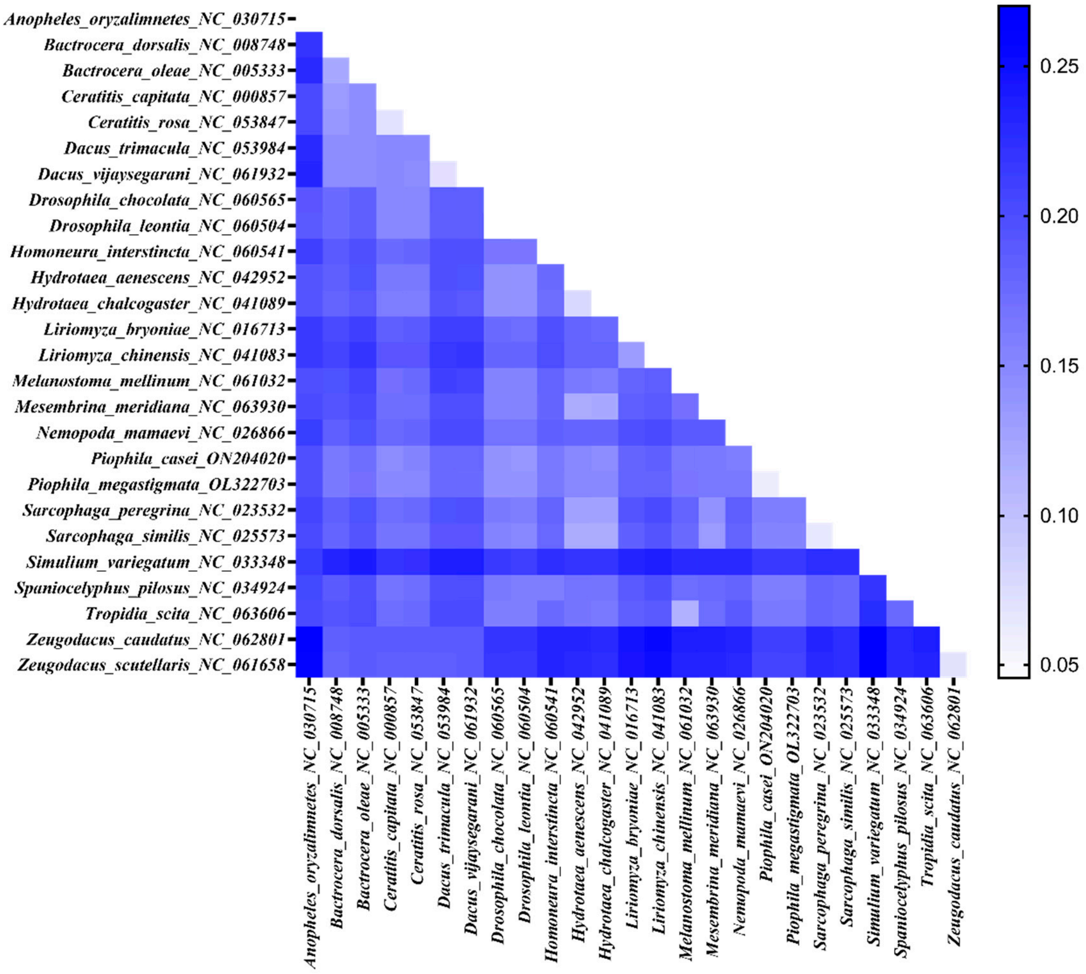

Figure S2. Genetic distances of mt genomes between 26 Diptera species

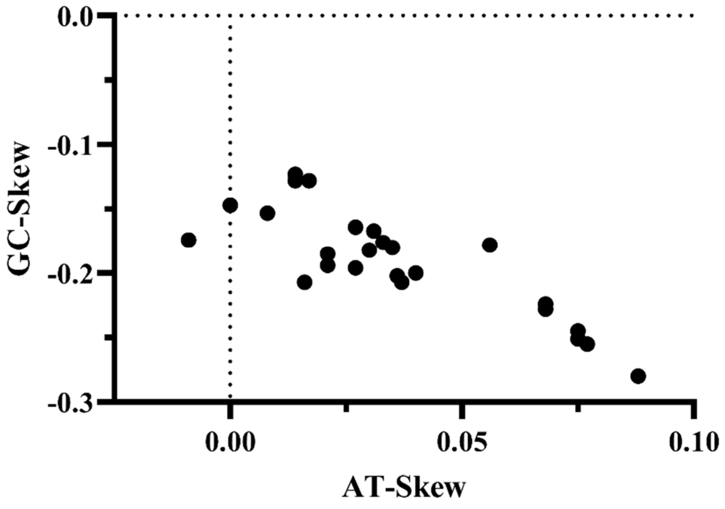

Figure S3. AT-Skew and CG-Skew values for the mt genomes of 26 Diptera species.

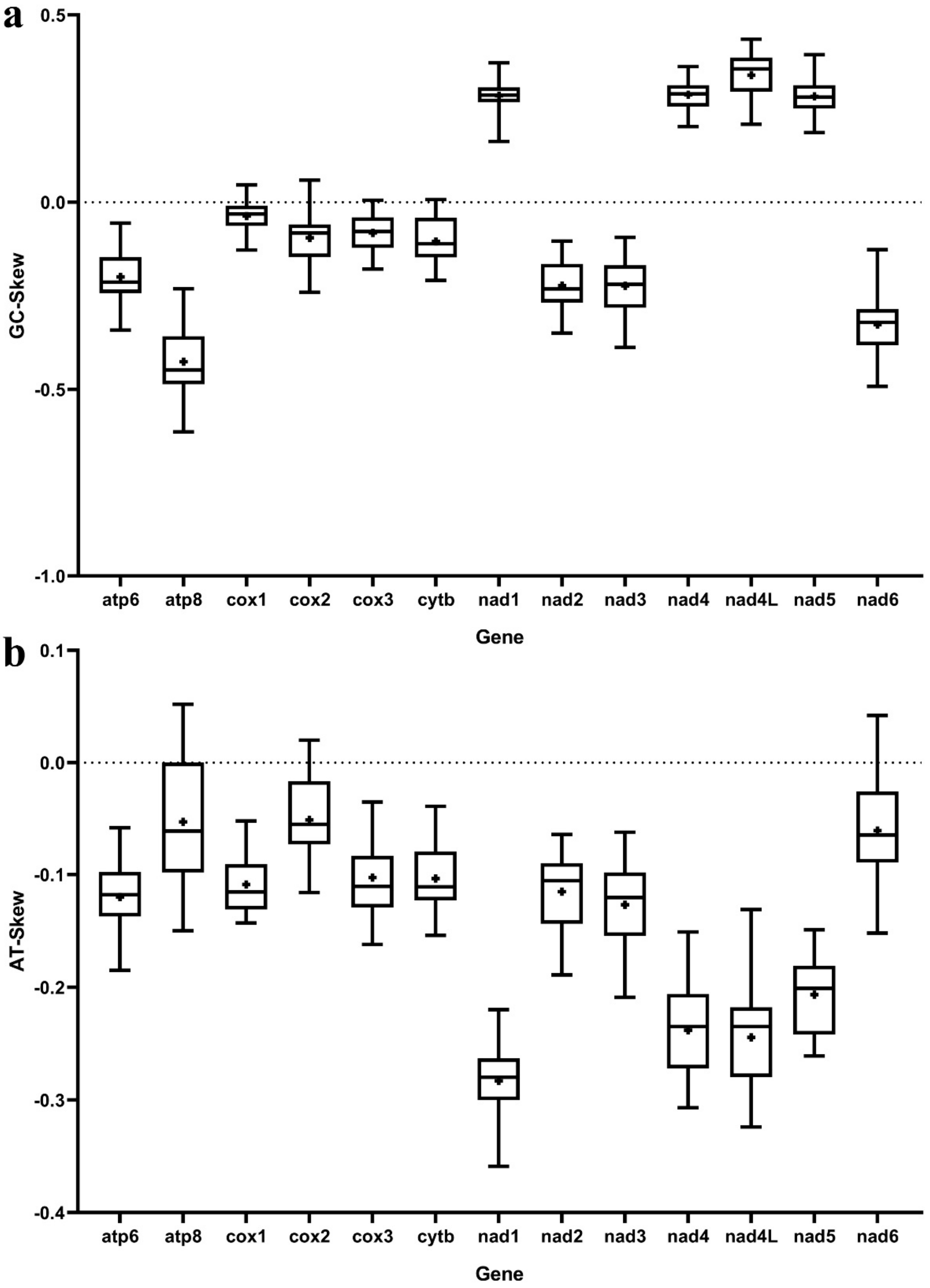

Figure S4. Box-and-whisker plots for nucleotide composition of each gene. (a)GC-skew; (b)AT-skew.

Table S2. Divergence time estimates calculate information such as the ESS of the resulting value.

Statistic	Mean	ESS	Type
joint	-1.629E5	1295	R
prior	-549.349	1139	R
likelihood	-1.623E5	1162	R
treeModel.rootHeight	245.193	1288	R

treeLength	2788.399	739	R
tmrca(1)	236.1	3193	R
tmrca(2)	153.888	911	R
yule.birthRate	9.874E-3	5909	R
gtr.rates.rateAC	0.243	520	R
gtr.rates.rateAG	1.79	228	R
gtr.rates.rateAT	0.648	339	R
gtr.rates.rateCG	0.594	428	R
gtr.rates.rateCT	2.494	300	R
gtr.rates.rateGT	0.232	368	R
frequencies1	0.367	272	R
frequencies2	9.45E-2	288	R
frequencies3	9.732E-2	463	R
frequencies4	0.441	335	R
alpha	0.244	518	R
pInv	0.446	1018	R
clock.rate	1	-	*
meanRate	1	-	*
treeLikelihood	-1.623E5	1162	R
branchRates	0E0	-	*
speciation	-143.439	2832	R

Table S3. Summary of the representative species and their mitogenome information in this study.

Family	Species	Accession no.	Size	AT
Tephritidae	*Ceratitis capitata*	NC000857	15,980	77.5
Tephritidae	*Bactrocera oleae*	NC005333	15,81515	72.6
Tephritidae	*Bactrocera dorsalis*	NC008748	15,915	73.6
Tephritidae	*Ceratitis rosa*	NC053847	16,047	77.4
Tephritidae	*Dacus trimacula*	NC053984	15,847	72.8
Tephritidae	*Dacus vijaysegarani*	NC061932	15,886	73
Tephritidae	*Zeugodacus scutellaris*	NC061658	15,931	73.1
Tephritidae	*Zeugodacus caudatus*	NC062801	15,311	72.7
Piophilidae	*Piophila megastigmata*	OL322703	15,410	76.4
Piophilidae	***Piophila casei*	ON204020	15,785	76.6
Syrphidae	*Tropidia scita*	NC063606	15,739	79.5
Syrphidae	*Melanostoma mellinum*	NC061032	16,055	81.2
Sepsidae	*Nemopoda mamaevi*	NC026866	15,878	74.8
Agromyzidae	*Liriomyza bryoniae*	NC016713	16,183	79.3
Agromyzidae	*Liriomyza chinensis*	NC041083	16,175	78.3

Sarcophagidae	*Sarcophaga peregrina*	NC023532	14,922	75
Sarcophagidae	*Sarcophaga similis*	NC022573	15,158	76.3
Muscidae	*Hydrotaea chalcogaster*	NC041089	15,279	78.6
Muscidae	*Hydrotaea aenescens*	NC042952	15,238	78.4
Muscidae	*Mesembrina meridiana*	NC063930	15,612	78.5
Celyphidae	*Spaniocelyphus pilosus*	NC034924	16,426	76.9
Lauxaniidae	*Homoneura interstincta*	NC060541	16,3511	75.6
Drosophilidae	*Drosophila leontia*	NC060504	16,019	75.3
Drosophilidae	*Drosophila chocolata*	NC060565	16,019	76.7
Culicidae	*Anopheles oryzalimnetes*	NC030715	15,422	77.7
Simuliidae	*Simulium variegatum*	NC033348	15,367	72.9

** The sequences for the species with names in bold were generated in this study.

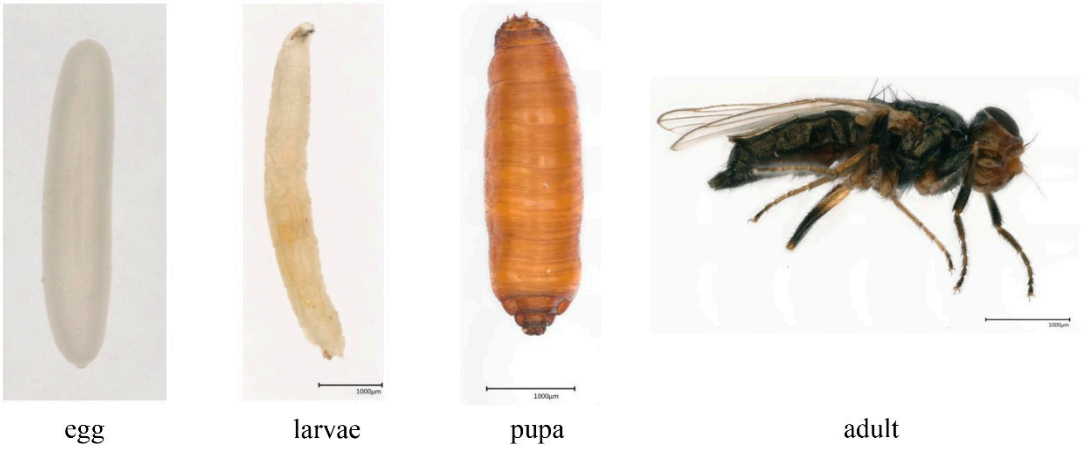

Figure S5. The life cycle states of *Piophila casei*.
